# The abundance of arbuscular mycorrhiza in soils is linked to the total length of roots colonized at ecosystem level

**DOI:** 10.1371/journal.pone.0237256

**Published:** 2020-09-11

**Authors:** Milagros Barceló, Peter M. van Bodegom, Leho Tedersoo, Nadja den Haan, G. F. (Ciska) Veen, Ivika Ostonen, Krijn Trimbos, Nadejda A. Soudzilovskaia

**Affiliations:** 1 Environmental Biology Department, Institute of Environmental Sciences, Leiden University, Leiden, Netherlands; 2 Natural History Museum, University of Tartu, Tartu, Estonia; 3 Department of Terrestrial Ecology, Netherlands Institute of Ecology, Wageningen, The Netherlands; 4 University of Tartu, Institute of Ecology and Earth Sciences, Tartu, Estonia; University of California Berkeley, UNITED STATES

## Abstract

Arbuscular mycorrhizal fungi (AMF) strongly affect ecosystem functioning. To understand and quantify the mechanisms of this control, knowledge about the relationship between the actual abundance and community composition of AMF in the soil and in plant roots is needed. We collected soil and root samples in a natural dune grassland to test whether, across a plant community, the abundance of AMF in host roots (measured as the total length of roots colonized) is related to soil AMF abundance (using the neutral lipid fatty acids (NLFA) 16:1ω5 as proxy). Next-generation sequencing was used to explore the role of community composition in abundance patterns. We found a strong positive relationship between the total length of roots colonized by AMF and the amount of NLFA 16:1ω5 in the soil. We provide the first field-based evidence of proportional biomass allocation between intra-and extraradical AMF mycelium, at ecosystem level. We suggest that this phenomenon is made possible by compensatory colonization strategies of individual fungal species. Finally, our findings open the possibility of using AMF total root colonization as a proxy for soil AMF abundances, aiding further exploration of the AMF impacts on ecosystems functioning.

## Introduction

Arbuscular mycorrhizal fungi (AMF) are widespread obligate symbionts forming associations with 85% of the vascular plant species [1], dominating most of the tropical forest and temperate grassland ecosystems [2, 3]. Besides the fundamental role of AMF in plant nutrition and fitness [4, 5], it is widely recognized that AMF have a substantial impact on ecosystem functioning. To understand this role, it is important to distinguish between the “intraradical mycelium”, which is the fungal biomass inside the root and the “extraradical mycelium” which is the fungal body in soil [6]. While the intraradical part will likely only affect ecosystem processes indirectly through host plant nutrition and performance [7], the extraradical mycelium is directly related to ecosystem functioning. AMF extraradical mycelium can modify the soil microbial community structure and composition [8–11], and enhance soil aggregation via stabilization of soil aggregates [12, 13]. The extraradical mycelium also acts as an active distributor of carbon (C) in the soil, feeding soil heterotrophs [14, 15] and stabilizing C in recalcitrant organic compounds [16, 17].

A comprehensive understanding of the impacts of AMF in the above-mentioned processes and the incorporation of mycorrhizal pathways into biogeochemical models requires quantitative measurements of AMF abundances in both of their functional compartments, roots and soils [18]. Information about AMF abundances in roots (typically expressed as percentage root length colonized) is common in the literature [e.g. 19–21]. In contrast, and despite their direct impact on C and nutrient cycling [7, 22, 23], the abundance of AMF extraradical mycelium in natural ecosystems is rarely reported and its relation to abundances of AMF in the plant roots is poorly understood.

So far, information based on studies of single fungal isolates indicates a general increase of AMF extraradical mycelium during the process of root colonization by AMF [24, 25]. This suggests that within the same single AMF species isolate, a higher intraradical C allocation generally leads to a higher C allocation in the soil compartment. However, natural ecosystems comprise a heterogeneous network of AMF species that may have remarkable differences in the proportion of biomass they allocate inside and outside the roots [26–28]. Laboratory studies demonstrate that, for instance, members of the AMF family Glomeraceae (order Glomerales) are known to have high intraradical colonization but only little expansion into the soil; members of Gigasporaceae (order Diversisporales) have the opposite colonization strategy and members of Acaulosporaceae (order Diversisporales) have low levels of both soil and root colonization [28]. Therefore, in a natural ecosystem where different AMF colonization strategies potentially coexist, whether an increase of AMF colonization in roots results in an increase of AMF mycelium in the soil is less evident and remains unsolved. Obtaining a field-based quantitative answer to this question will 1) provide important insights into the mechanisms of C and nutrient flow through mycorrhizal pathways at ecosystem level, and 2) will inform us about the feasibility of using estimates of AMF abundance in roots as a proxy of AMF soil abundance.

**Fig 1:**
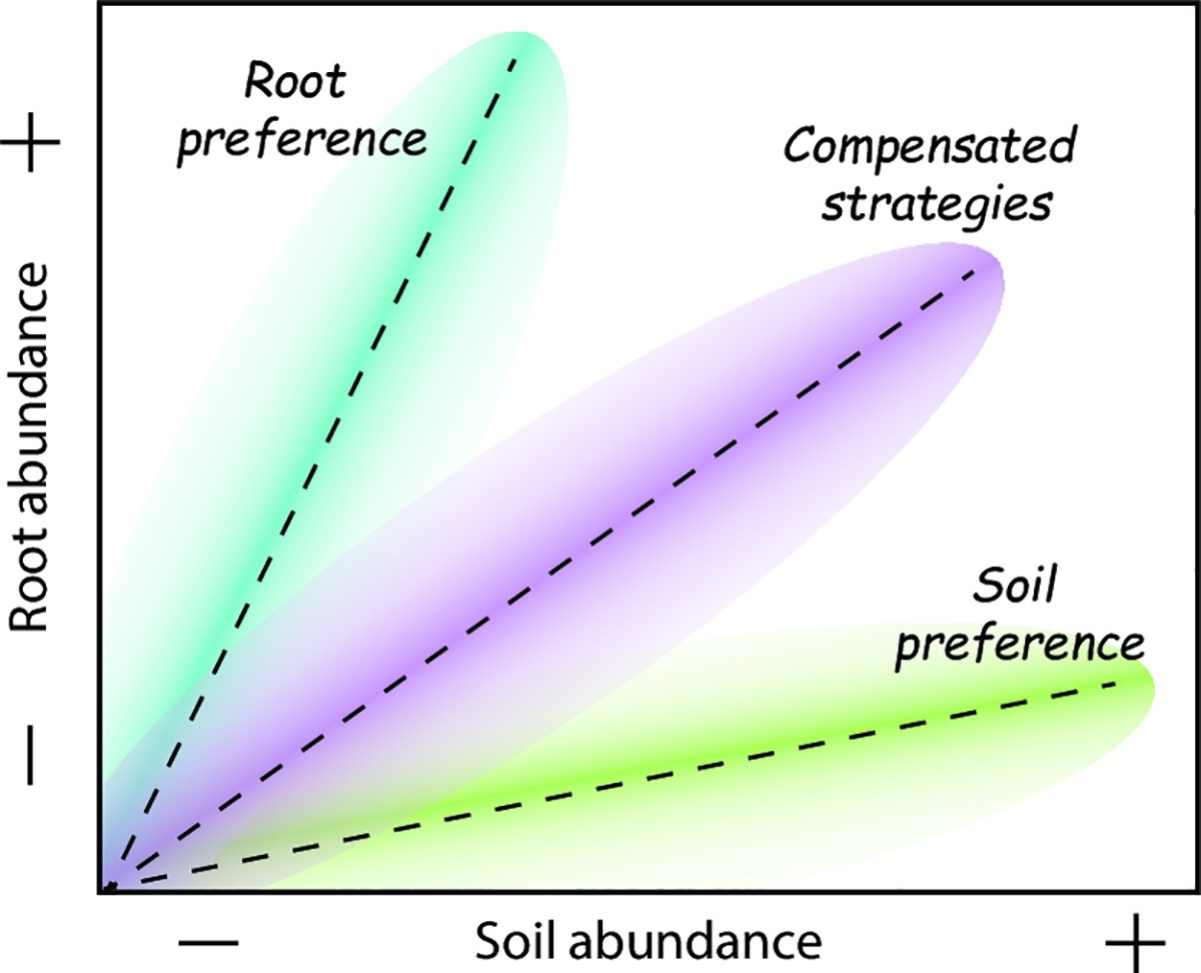
Conceptual scheme indicating possible patterns between the abundance of AMF in roots vs soil compartments. Dotted lines and coloured shapes represent three different scenarios depending on the predominance of AMF taxa with different colonization strategies. A first scenario (green shape) represents an AMF community dominated by species with preference for soil colonization. A second scenario (blue shape) represents an AMF community dominated by species with a preference for root colonization. In a third scenario (purple shape) both root and soil colonization strategies are present but their abundance tends to even out. Finally, a fourth scenario where no relationship is expected (not shown in the graph) if a) community assembly along the plant community is random (no compensated colonization strategies) or if b) the biomass allocation in root and soil of individual AMF taxa is not coupled.

Here, we explore the quantitative patterns of AMF abundances in roots vs soil and the corresponding community composition in a natural dune grassland to answer the following questions: 1) is the level of colonization by AMF in the roots positively related to the abundance of AMF mycelium in the soil, within a natural ecosystem? And if so, 2) do different AMF colonization strategies influence the relationship between AMF abundance in root and soil compartments? We hypothesize that, if at the ecosystem level a single colonization strategy dominates, the proportion of biomass allocated by AMF in roots and soil compartments will remain constant, and therefore a relationship between the biomass in soil vs roots may be expected. If root and soil colonization strategies co-occur along the plant community but their intra- and extraradical relative abundances are complementary, a correlation between root and soil AMF biomass may also be expected (see Fig 1).

The intensity of plant root colonization by mycorrhizal fungal is typically expressed as percent of root length (AM) or root tips (ECM) colonized by fungi. Higher intensities of root colonizetion by AM fungi are associated with greater mycorrhizal dependency of the host plant (measured as a relative increase in biomass of a plant in the presence of mycorrhizae) (Wilson & Hartnett, 1998; Treseder, 2013)

## Methods

### 1. Sample collection

Plant and soil samples were collected in May 2017 in the Kennemer Dunes National Park (52.43 N, 4.57 E), a 25 km^2^ dune ecosystem situated along the north coast of the Netherlands. Based on a visual inspection of vegetation conditions to avoid non-mycorrhizal plants, a 350-meter-long transect was established, covering a gradient from moist to dry soils. Such natural moisture gradient was used as a mean to ensure sampling of plant communities featuring distinct levels of AMF root colonization. This expectation is based on the fact that AMF are suppressed by high soil moisture [29, 30]. Within this transect, fifteen sampling points were established. Areas with known non-mycorrhizal species were avoided.

At each sampling point, we established a circular area of approximately 3 m diameter, where five subsamples, separated from each other by at least 1 m, were collected from the topsoil layer (15 cm). These subsamples were later pooled and homogenized to obtain a total volume 1 dm^3^ of soil. During the collection, samples were kept frozen using dry ice to avoid degradation of organic compounds.

From each sample, soils and roots were separated by sieving. The extracted roots were carefully cleaned with tap water and weighted. Half of the root samples were preserved in 50% ethanol for AMF colonization measurements while the rest was oven-dried (35°C, 30 h) for molecular analysis.

### 2. Root colonization

To estimate AMF root colonization, roots preserved in ethanol were first cut into small pieces (ca 1 cm each), cleaned with 2.5% KOH and stained by autoclaving in 5% Pelikan Blue ink [31]. The percentage of colonization was estimated by examining vesicles, hyphae and arbuscular structures with a grid line intersect procedure [32]. Total root length was measured with the WinRhizoTM Pro 2003b image analysis system (at 400 dpi; Regent Instruments Inc., Ville de Québec, QC, Canada). Standing root length colonized by AMF (used as a proxy of AMF abundance in roots) was calculated by multiplying the percentage of colonization and total root length per volume of soil.

### 3. Extraradical mycelium abundance

The abundance of AMF extraradical mycelium was measured using fatty acid analysis. The lipid extraction from 3g of freeze-dried soil was performed using a one-phase mixture following Bligh and Dyer [33] and modified by Frostegård et al [34]. The neutral lipid fatty acid (NLFA) 16:1ω5 was used as a proxy for AMF abundance [35, 36].

### 4. AMF community structure

DNA was extracted following the protocol of Tedersoo et al [37] using a PowerSoil DNA Isolation Kit (Mo Bio Laboratories, Inc., Carlsbad, CA, USA). We used 0.25 g of dried soil and 0.1 g of ground dried roots. Polymerase chain reaction (PCR) was performed using the primer pair ITS9mun/NS8a [38] targeting the rRNA 18S gene V9 variable region. This universal primer set was selected to cover most of the fungi including phylum *Glomeromycota* across an intron-free fragment of equal length [39]. The PCR program consisted of 15 min incubation at 95°C, followed by 25 cycles of 30 s at 95°C, 30 s at 55°C and 50 s at 72°C. PCR products were purified using Favorgen GEL/PCR Purification Mini Kit. Amplicons were sequenced with Illumina MiSeq platform at the Estonian Genome Center.

Sequencing data were analyzed with PipeCraft [40]. To remove low-quality reads, filtering was performed with vsearch (v1.11.1) (parameters: minoverlap = 15, minlength = 50, E_max = 1, maxambigu = 0). Operational Taxonomic Units (OTU) were constructed using the UPARSE algorithm [41] at 97% sequence similarity threshold. Singleton clusters were removed. A post-clustering curation to OTU table was performed with LULU [42]. Representative OTU sequences were taxonomically assigned using SILVA (release 128) database [43] with BLAST [44] (threshold criterion e-value<e^-10^). Chimera check was performed using UCHIME de novo option. No rarefaction was done because richness was unrelated to sequencing depth. Raw Illumina data sets have been deposited in the Sequence Read Archive (SRA) under BioProject PRJNA644291. The resulting OTU table was deposited in Figshare public repository (https://doi.org/10.6084/m9.figshare.9785930.v1).

### 5. Statistical analysis

The relationship between root length colonization and NLFA abundances was assessed using a linear model for vesicles, arbuscules and hyphae separately. Differences between root and soil AMF community structures were visualized using non-metric multidimensional scaling (NMDS) with Bray–Curtis dissimilarity using “metaMDS” function in R Package “vegan”. Root and soil community differences were tested for statistical significance using permutational multivariate analysis of variance (PERMANOVA) (“adonis” function in R Package “vegan”). PERMANOVA’s assumption of homogeneity in within-groups variability was tested using “betadisper” function in R Package “vegan”. All statistical analyses were performed using R 3.4.3 [45].

## Results

### 1. Relationship between intra-and extraradical mycelium

**Fig 2:**
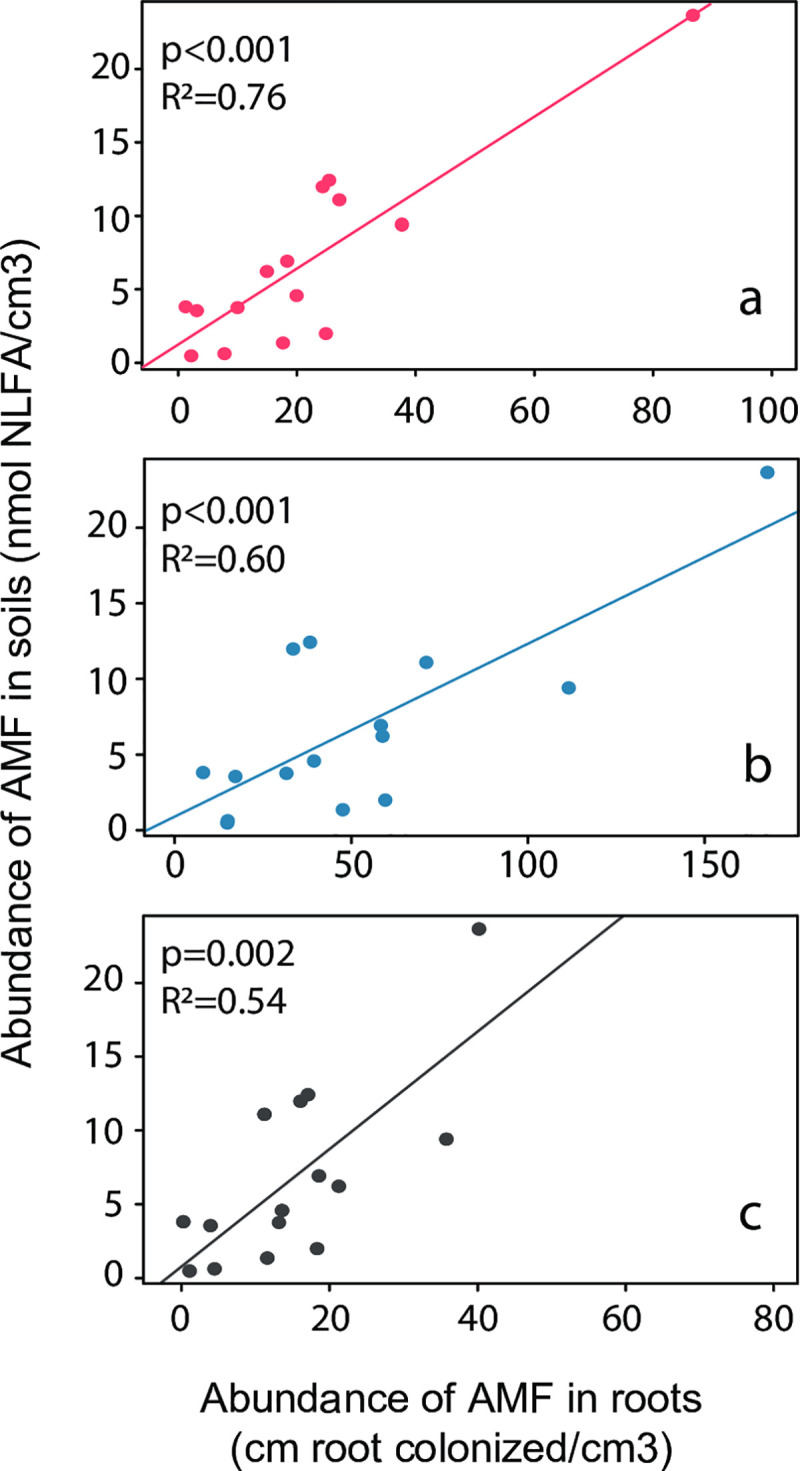
Linear relation between the AMF biomass in the soil and the total root length colonized for the three detected AMF structures. (a) arbuscules (b) hyphae and (c) vesicle. NLFA 16:1w5 was used as a proxy of the AMF biomass in the soil.

The linear regression models showed a significant positive relationship between the amounts of NLFA 16:1w5 in the soil and total root length colonization at the studied community (Fig 2). This positive relation was consistent among the different AMF structures for which colonization was measured (arbuscular, hyphae and vesicles). Root colonization by arbuscular structures showed the strongest relation with NLFA 16:1w5, followed by hyphal colonization and vesicle colonization. Despite the influence of an extremely high NLFA 16:1w5 value, the relation remained highly significant when this value was removed from the input data (see Fig S1 in S1 Appendix).

### 2. Community composition

**Fig 3:**
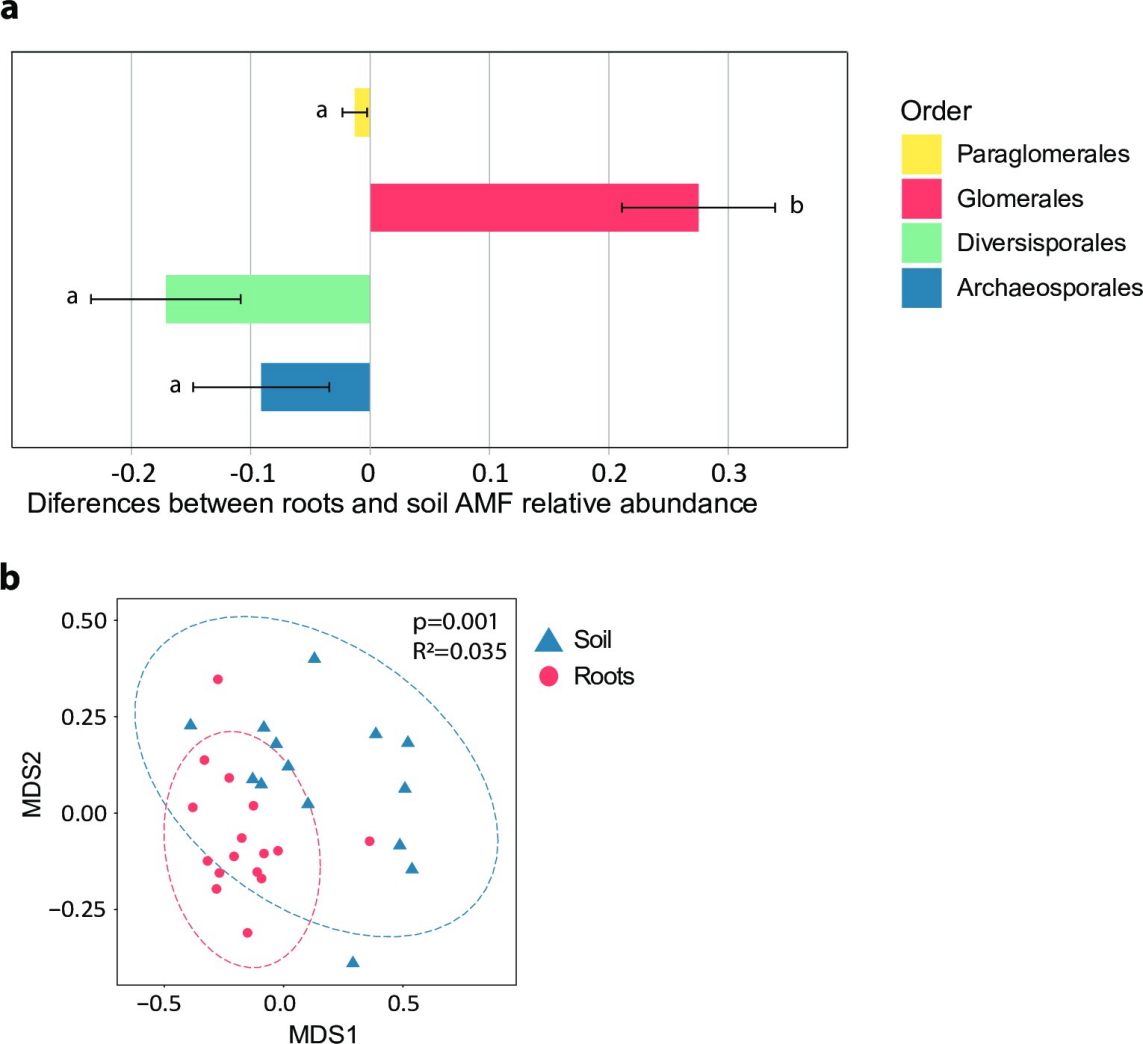
Differences in AMF community composition between root and soil samples. a) Means and standard error of the difference between the relative abundances of the AMF orders Archaeosporales, Diversisporales, Glomerales and Paraglomerales in soil and root samples. Positive values indicate that, on average, root samples had a higher relative proportion than their soil pairs, while negative values indicate the opposite trend. Different letters indicate significant differences between orders. b) Nonmetric multidimensional scaling (NMDS) ordination plots of arbuscular mycorrhizal fungal communities present in soil and roots compartments based on relative proportions of OTUs. Ellipses delimit the 95% confidence interval around centroids.

Community composition analysis showed a clear dominance of members of the order Glomerales in root samples (see Fig S2 in S1 Appendix). In contrast, soil samples showed a more heterogeneous composition, having in general a higher proportion of the order Diversisporales, Archaeosporales and Paraglomerales than in root samples (Fig 3A). Therefore, a general shift in the relative abundance of the four Glomeromycota orders can be seen between soil and plant roots. PERMANOVA analysis ratified this pattern showing significant differences (*R*^2^ = 0.035, *p* = 0.001) in AMF community composition between soil and roots based on OTUs’ relative abundances (Fig 3B).

## Discussion

### 1. Relationship between root vs soil AMF abundance

We found a strong positive relationship between the abundance of AMF in soil and the total root length colonized. Although a similar pattern has been found before in single AMF isolates [24, 25], our results provide the first evidence of a relationship between intra- and extraradical AMF abundance at an entire plant community level in a natural ecosystem. This suggests that, even at plant community level where different AMF species are expected, plant C allocation to the symbiotic fungi is proportionally distributed between root and soil compartments.

The strong relationship found here raises the question of whether measurements of total root colonization can be used to infer information about AMF abundance in soils or vice versa. Given the relevance of AMF for ecosystem functioning, this link is promising to gain understanding of AMF distribution in soils and roots. However, the extrapolation of the patterns found in this study to different ecosystems and environmental conditions requires caution and further testing.

Firstly, while the techniques applied here (fatty acid analysis and microscopic quantification) are widely used, they are known to induce serious biases [46, 47] that may introduce uncertainties to our results. Therefore, within the framework of this research, we have explored the possibility to use molecular quantification tools (i.e. the novel digital droplet PCR technique [48]) as a potentially robust, accurate and rapid methodological alternative to assess AMF abundance in roots and soil. This test and its outcomes are presented in detail in the S2 Appendix. In short, we have detected that the abundance of AMF in roots using ddPCR was positively related with the total root length colonized, while using ddPCR for examining the abundance of AMF in soil was problematic, and delivered obscured results. We conclude that ddPCR techniques can already be used for the assessment of AMF abundance in roots, while the methods of using this technique for soil samples still need further development (see S2 Appendix for recommendations).

A second potential source of uncertainties it that neither the traditional nor the molecular techniques explored here can discriminate between active and dormant stages or recently dead biomass [49]. This issue is potentially less problematic when our results are used in the context of nutrients and C cycling assessments. However, it should be considered when the assessment of ecosystem function is the main goal and differences in microbial physiological states are relevant.

### 2. Role of community composition

A crucial step towards further generalizations of the relationship found here is disentangling the role of AMF community composition and, specifically, the contribution of different colonization strategies (i.e. soil vs root colonizers) in affecting the relationship between root and soil abundances.

In line with the findings of several field experiments [50–52], we found that AMF community composition differed between roots and soil compartments (Fig 3B). While roots were clearly dominated by members of the order Glomerales (Fig S2 in S1 Appendix), their relative abundance in soil samples tended to decrease, being partially replaced by members of the order Archeaeosporales and Diversisporales (Fig 3A). This shift in community composition between root and soil reflects, as proposed by Hart and Reader [28], differences in colonization strategies among the main AMF groups.

Despite these differences in community composition, the ratio between AMF biomass in root and soil compartments remains relatively constant, as reflected by the strongly significant linear relationship of soil vs roots abundance (Fig 2). This suggests that the second theoretical scenario presented in Fig 1 prevails in the studied system, indicating that co-occurring AMF species have compensatory colonization strategies, resulting in a robust relationship between soil and root abundances. The co-occurrence of different strategies may reflect an ecological specialization of co-existing AMF linages to avoid competition for space and resources [53–55]. Moreover, different colonization strategies have been proposed to relate to different benefits to the plant. A more extensive extraradical mycelium is generally associated with an increase of nutrients supply to the plant [56], while a higher intensity of root colonization provides the host plant with greater protection against soil pathogens [57]. Therefore, the C flow from the host plant to their fungal partner may be distributed within different functional strategies to maximize fitness [58], which ultimately leads to coupled AMF abundances inside and outside the plant roots, even at the community level.

Even though colonization strategies seem to play an important role in assembling AMF communities, other environmental factors such as soil properties [59] or plant identity [60]) can also influence AMF community composition. Within an ecosystem, the chance that higher AMF abundance in roots leads to a higher abundance in the soil will ultimately depend on the relative contribution of distinct AMF functional groups to the intra- and extra-radical biomass. Therefore, in particular conditions specific strategies may be favored, affecting the relations found here. We tested if deviations in the AMF community mean composition of the order Divesisporales (chosen as a reference group due to its higher relative abundance in soils) were related to deviations in the relationship of AMF abundance between soil and roots (Fig S3 in S1 Appendix). We found that if in a given location, the relative abundance of the order Divesisporales was higher than the community mean, the abundance of AMF in the soil was underestimated by the linear model (as indicated by positive residuals in the abundance correlation). This suggests that plant communities within which the AMF colonization strategies are not fully evened out, the relationship between intra- and extraradical may be weakened.

Further research targeting the absolute abundance of specific groups with different colonization strategies will be key to improve our understanding of AMF community assembly rules and its role in the abundance pattern of soils vs roots.

## Conclusions

Our results provide the first direct evidence of a relation between AMF abundances in soils and roots at the ecosystem level, suggesting that the input from the host plant is proportionally distributed between the root-associated mycelium and the extraradical mycelium. This relationship of AMF abundances is likely to be caused by compensatory colonization strategies of individual fungal species. Specific environmental conditions may favour certain functional groups that may interfere with the coupling of AMF abundances at community level, which will demand further testing. Our findings open the possibility of using AMF intraradical abundance measurements as a proxy of extraradical abundance at a community scale. This proxy will help to estimate AMF abundance in soils, which is key towards a better understanding of terrestrial ecosystems functioning in present and future climates.

## Supporting information

S1 Appendix (DOCX)

S2 Appendix (DOCX)
