# Supplementary material for: The abundance of arbuscular mycorrhiza in soils is linked to the total length of roots colonized at ecosystem level
Source: PLoS One. 2020 Sep 11;15(9):e0237256. doi: 10.1371/journal.pone.0237256 (PMC7485760; doi:10.1371/journal.pone.0237256)
Supplement: S1 Appendix — (DOCX) [file pone.0237256.s001.docx]

**Appendix S1: Supplementary Figures**


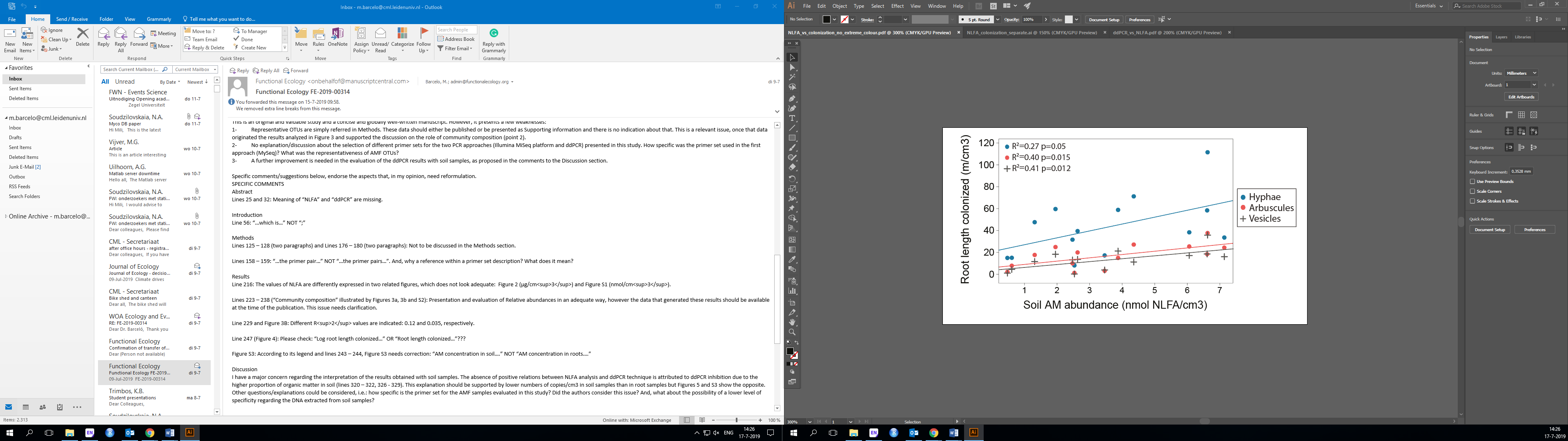


**Fig S1. Linear relation between the AMF biomass in the soil and the total root length colonized for the three detected AMF structures.** NLFA 16:1w5 was used as a proxy of the AMF biomass in the soil. *One extreme value of NLFA (23.65 nmol/cm3) was omitted, compared to the relationship shown in Fig. 2 in the main text, to evaluate the influence of this point on the significance of the relationship.*


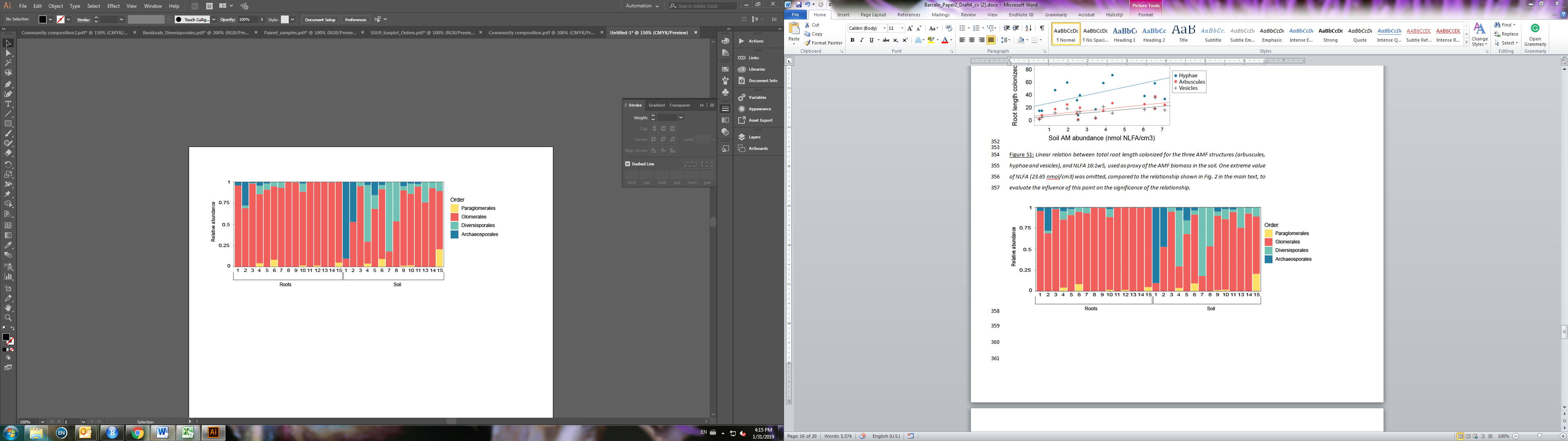


**Fig S2. *Relative abundance of the main AMF orders in roots and soil in each sample location.***


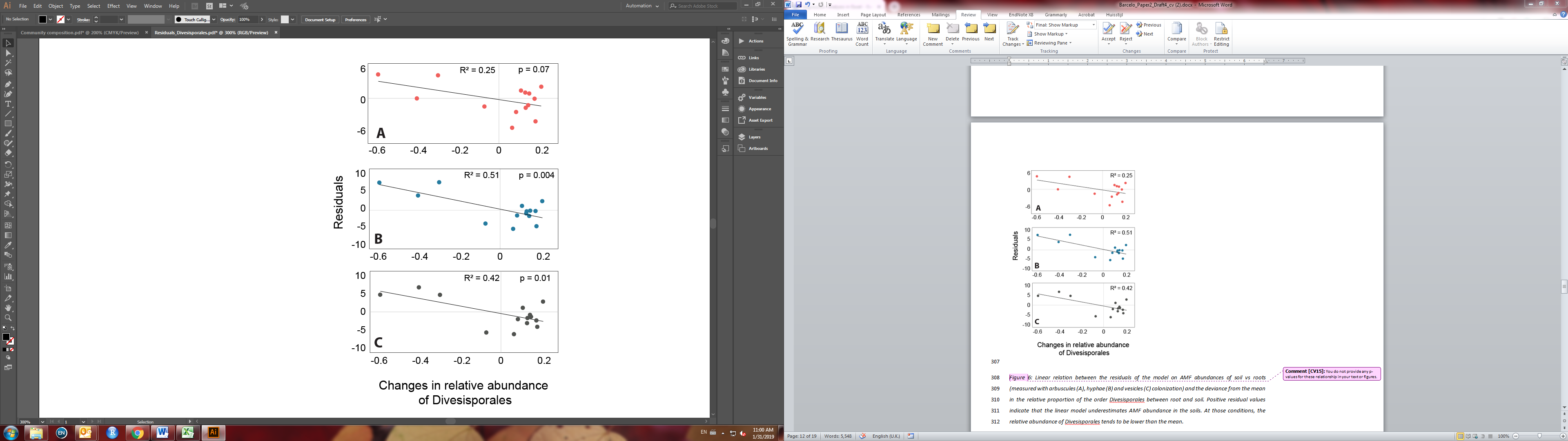


**Fig S3. *Linear relation between the residuals of the model on AMF abundances of soil vs roots (measured with arbuscules (A), hyphae (B) and vesicles (C) colonization) and the deviance from the mean in the relative proportion of the order Divesisporales between root and soil****. Positive residual values indicate that the linear model underestimates AMF abundance in the soils. At those conditions, the relative abundance of Divesisporales tends to be lower than the mean.*
