# Supplementary material for: The abundance of arbuscular mycorrhiza in soils is linked to the total length of roots colonized at ecosystem level
Source: PLoS One. 2020 Sep 11;15(9):e0237256. doi: 10.1371/journal.pone.0237256 (PMC7485760; doi:10.1371/journal.pone.0237256)
Supplement: S2 Appendix — (DOCX) [file pone.0237256.s002.docx]

**Appendix S2: Evaluation of AMF abundance in roots and soils using digital droplet PCR (ddPCR)**

As traditional method may induce considerable biases, we tested a new, potentially more reproducible technology, ddPCR.

1. Methods

Intraradical and extraradical abundance of AMF was complementarily assessed using a digital droplet PCR (ddPCR) analysis. This technique provides an absolute quantification of target DNA copy number by partitioning the PCR reaction in multiple replicates (droplets) and detecting successful DNA amplification in each replicate (Hindson et al., 2011). The ddPCR technique is more accurate and sensitive than the commonly used quantitative PCR (qPCR), especially at low DNA concentrations (Hindson et al., 2013; Yang et al., 2014; Doi et al., 2015; Nathan et al., 2015). Furthermore, the fact that ddPCR analysis does not need calibration curves nor many replicates makes it a more cost-efficient alternative to qPCR (Doi et al., 2015b; Hindson et al., 2013; Nathan et al., 2014; Yang et al., 2014).

Quantitative molecular techniques require the use of specific primers that avoid amplifying non target organisms. For this reason, we used the reverse primer AM1 (Helgason et al., 2002) and the forward AMG1F (Hewins et al., 2015) that specifically target members of the filum Glomeromycota. The final ddPCR reaction volume of 22 μl contained 2 μl DNA extract, 2.5 μl 100 nM forward and reverse primers, 10 μl Bio-Rad Evagreen Supermix (Bio-Rad, Hercules, CA, USA) and 5 μl Milli-Q. Of this 22 μl PCR mixture, 20 μl were transferred onto a DG8 Biorad cartridge containing 8 wells, of which one was used for a blanc sample containing 3 μl Milli-Q. Droplets were produced using a Bio-Rad QX-200 droplet generator with 70 μl Bio-Rad generator oil per well. Of the resulting emulsion mixture, 40 μL of the produced droplet mixture was pipetted into a semi skirted twintec 96-well plate and sealed using the PX1 PCR Plate Sealer (Bio-Rad). The ampliﬁcation program incorporated an initial 95 °C denaturation for 5 min, followed by 35 cycles of 15 s at 95 °C, 60 s at 62 °C, and 90 s at 72 °C and a final step of 90 s at 72 °C. The samples were then analyzed with a QX200 Droplet Reader and processed with QuantaSoft software version 1.7.4 (Bio-Rad) to obtain AMF copy number/ μl of PCR mixture. Droplets were assigned as positive or negative by thresholding based on the height of their respective fluorescence amplitude. The absolute DNA concentration was estimated from the proportion of total positive reactions and the initial concentration of the sample using a Poisson distribution. Roots and soil results were transformed to AMF copy number/cm3 of soil.

1. Results

We found a significant positive relationship between the total number of DNA copies detected by ddPCR inside the root compartment and the total root length colonization measured by microscopic identification (Fig. S1). In contrast, we did not find a relation between ddPCR estimation in soils and NLFA 16:1w5 values (Fig. S2). Concomitantly, the total number of AMF DNA copies measured inside the roots was not significantly related to the number of AMF DNA copies in soils (Fig. S3).


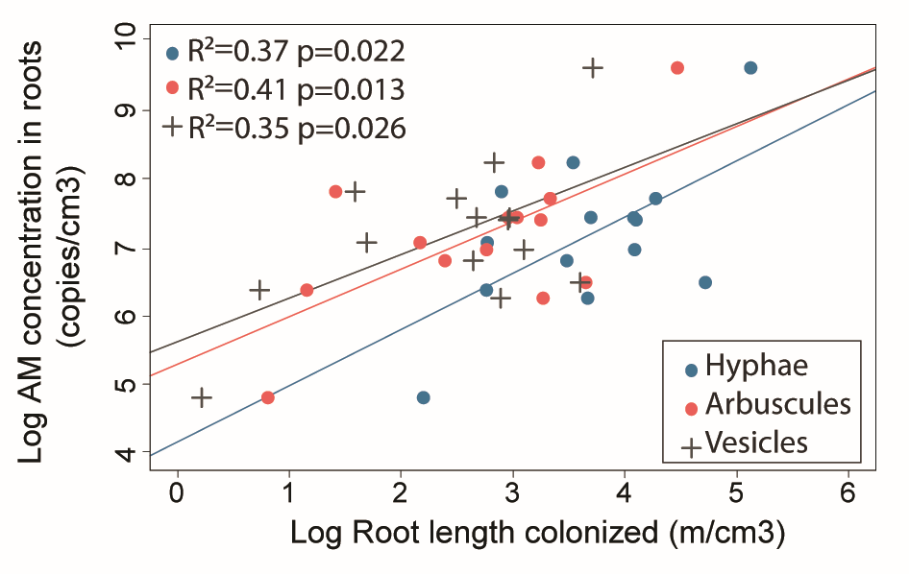


**Fig S1.** ***Linear relation between the abundance of AMF in roots measured with ddPCR and root length colonized by the three detected AMF structures (hyphae, arbuscules and vesicles).***


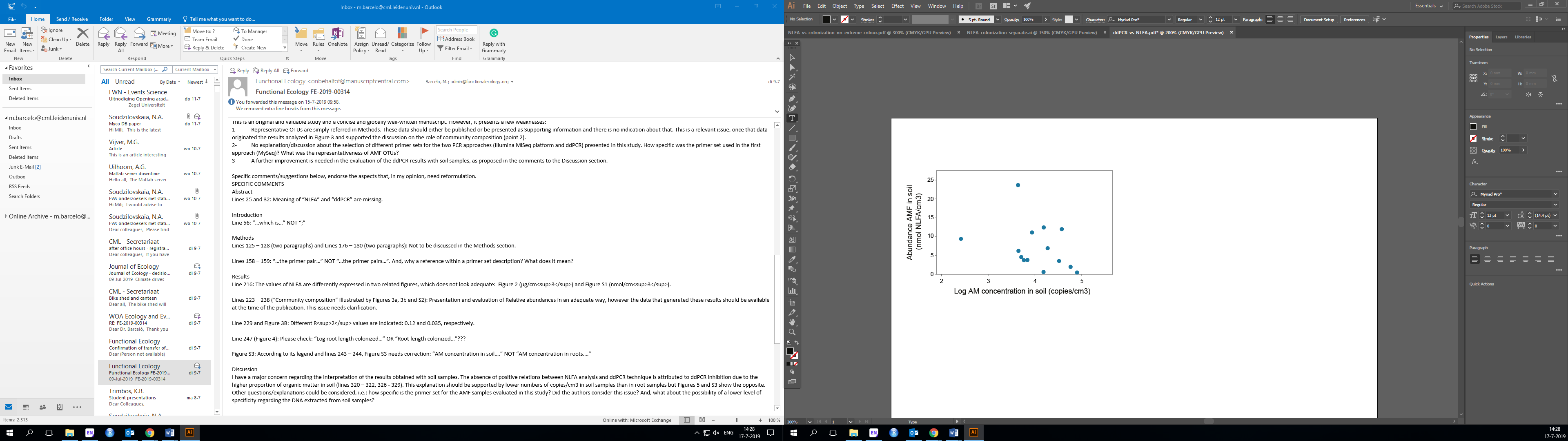


**Fig S2.** **C*orrelation between AMF abundances in soil measured with ddPRC technology and AMF abundance measured with fatty acids analysis.*** *The total number of DNA copies detected in the samples is used as proxy of AMF abundance.*


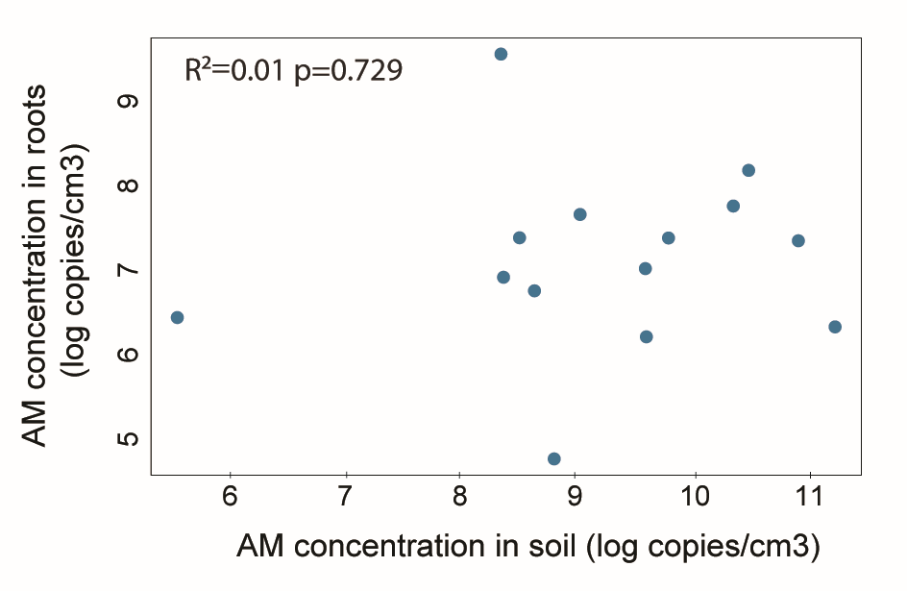


**Fig S3.** ***The relationship between AMF abundances in soil and roots compartments using ddPRC technology.*** *The total number of DNA copies detected in the samples is used as a proxy of AMF abundance.*

1. Discussion

The ddPCR technique has been shown to be a promising tool to quantify with high precision the abundance of target DNA molecules (Kim et al., 2014; Doi et al., 2015). Despite its potential, ddPCR has been rarely used in ecological research. Here, we tested the possibility to assess AMF abundance both in roots and soils using ddPCR. The abundance of AMF in roots using ddPCR was positively related with the total root length colonized (Fig. S1). This clearly reflects the applicability of ddPCR techniques for the assessment of AMF abundance in roots. However, it seems that the higher proportion of organic compounds in soil interfered with the PCR reaction, leading to a lack of positive relation between NLFA analysis and ddPRC technique in soil samples (Fig. S2). This interference was indicated by a much lower difference in fluorescence amplitudes between positive and negative observations in soil (even though ddPCR has been shown to be more robust to effects of organic compounds than other PCR techniques (Rački et al., 2014; Cavé et al., 2016)). Thus, the use of ddPCR for AMF quantification in soil samples requires further optimization and standardization of protocols that can mitigate the high concentration of PCR inhibitors. Possibly, inclusion of additional cleaning steps or using inhibitor removal kits such as the DNeasy PowerClean Pro Cleanup Kit (Qiagen) our protocols might yield better ddPCR results. The relatively poor performance of our ddPCR protocol in soil also seems to explain the lack of relationship between soil and root abundances when using ddPCR. Together, this implies tailor-made ddPCR protocols for soil samples are needed before it may replace the labor intensive, hard to automate and time-consuming traditional methodologies.
